# Enhanced flow-motion complexity of skin microvascular perfusion in Sherpas and lowlanders during ascent to high altitude

**DOI:** 10.1038/s41598-019-50774-0

**Published:** 2019-10-07

**Authors:** Deborah Carey, Marjola Thanaj, Thomas Davies, Edward Gilbert-Kawai, Kay Mitchell, Denny Z. H. Levett, Michael G. Mythen, Daniel S. Martin, Michael P. Grocott, Andrew J. Chipperfield, Geraldine F. Clough

**Affiliations:** 1 Faculty of Engineering and Physical Sciences, University of Southampton, Southampton, UK; 2 University College London Centre for Altitude Space and Extreme Environment Medicine, UCLH NIHR Biomedical Research Centre, Institute for Sports Exercise and Health, London, UK; 3 Integrative Physiology and Critical Illness Group, Clinical and Experimental Sciences, Faculty of Medicine, University of Southampton, Southampton, UK; 4 Faculty of Medicine, University of Southampton, Southampton, UK; 5 Respiratory and Critical Care Research Theme, Southampton NIHR Biomedical Research Centre, University Hospital Southampton NHS Foundation Trust, University of Southampton, Southampton, UK; 6 Anaesthesia and Critical Care Research Unit, University Hospital Southampton NHS Foundation Trust, Southampton, UK

**Keywords:** Blood flow, Biomedical engineering

## Abstract

An increased and more effective microvascular perfusion is postulated to play a key role in the physiological adaptation of Sherpa highlanders to the hypobaric hypoxia encountered at high altitude. To investigate this, we used Lempel-Ziv complexity (LZC) analysis to explore the spatiotemporal dynamics of the variability of the skin microvascular blood flux (BF) signals measured at the forearm and finger, in 32 lowlanders (LL) and 46 Sherpa highlanders (SH) during the Xtreme Everest 2 expedition. Measurements were made at baseline (BL) (LL: London 35 m; SH: Kathmandu 1300 m) and at Everest base camp (LL and SH: EBC 5,300 m). We found that BF signal content increased with ascent to EBC in both SH and LL. At both altitudes, LZC of the BF signals was significantly higher in SH, and was related to local slow-wave flow-motion activity over multiple spatial and temporal scales. In SH, BF LZC was also positively associated with LZC of the simultaneously measured tissue oxygenation signals. These data provide robust mechanistic information of microvascular network functionality and flexibility during hypoxic exposure on ascent to high altitude. They demonstrate the importance of a sustained heterogeneity of network perfusion, associated with local vaso-control mechanisms, to effective tissue oxygenation during hypobaric hypoxia.

## Introduction

Sherpas highlanders (SH) are known to demonstrate considerable tolerance to hypobaric hypoxia, however, the mechanisms behind this adaptation are not well understood^1^. Previous studies have shown that SH exhibit a lower arterial oxygen content than lowlanders (LL) who ascend to comparable altitudes^2–4^, and thus it has been suggested that the mechanisms that facilitate SH apparent hypoxia tolerance reside, in part, within the microcirculation^5–8^. Adaptive SH microcirculatory mechanisms that are not evident in LL include a sustained microvascular perfusion and dilator capacity measured in the skin^8,9^ and a higher microvascular flow index (MFI) and reduced perfusion heterogeneity index (HI) in the small (<25 µm diameter) vessels of the sublingual mucosa^7,8^.

Blood flow within a microcirculatory network is highly variable and its distribution and magnitude determined by local and regional metabolic demand. Local control of blood flow ensures matching of perfusion to spatially varying oxygen demand, thereby achieving efficient oxygen delivery^10,11^. Regulation of microvascular perfusion is predominately achieved through changes in network conductance, modulated at a local level by endothelial, neurogenic and myogenic regulatory activity^12^. Together, these activities determine the cyclic oscillations of arteriolar diameter (vasomotion) that are related to changes in blood flow distribution in the microvascular networks (flow-motion)^13^. The relative contribution of this rhythmic, low frequency oscillatory activity, as well as higher frequency cardiac and respiratory rhythms to microvascular perfusion has been assessed using spectral analysis of blood flux (BF) signals obtained using non-invasive laser Doppler fluximetry in the skin^14^. This has led to the suggestion that measurement of flow-motion activity may provide an early indicator of declining function (for review see^15^). In SH, the sustained network perfusion seen on exposure to hypobaric hypoxia has been associated with an enhanced low frequency flow-motion activity that was not evident in LL^8,9^. However, the extent to which such changes in local low-frequency oscillatory activity contribute to the observed variability in microvascular blood flow under conditions of hypobaric hypoxia^7,8^ remains unexplored; as does the extent to which such oscillatory activity may be beneficial to tissue oxygenation under such conditions^11,16,17^.

Nonlinear complexity measures have been extensively used to assess the variability or loss of randomness of bio-signals notably electroencephalograms^18^ and electrocardiograms^19^. The spatiotemporal variability of these signals reflect the physiological adaptability of the system and are established biomarkers of overall health status^20^. We and others have used non-linear analysis, such as Lempel-Ziv (LZ) complexity^21^, to determine the information content of microcirculatory blood flow signals as a metric of perfusion variability^22,23^. To date, these measures have confirmed a declining regularity and randomness of microcirculatory BF signals in both a primate model of diabetes^24^ and in humans with or at risk of cardiovascular disease (CVD) in whom microvascular dysfunction is evident^25^. To what extent the variability in the BF signals arising from the cumulative activity (and temporal variation) of all the processes modulating microvascular blood flow differs between SH and LL, and with ascent to altitude, has yet to be investigated; as does the impact of the complexity of the BF signal on effective tissue oxygenation.

The processes that regulate the cardiovascular system operate across multiple temporal scales. Consequently, multiscale nonlinear analysis of complex oscillatory signals such as heart rate variability has been used, in addition to traditional time- and frequency-domain analysis, as a tool to enhance diagnosis and risk stratification^25^. The rhythmical oscillatory processes that determine flow-motion, and that operate across differing time-scales in the range ~0.6–100 seconds, have similarly been explored using multiscale non-linear analysis of the laser Doppler BF signal^22,23,26^. To date these studies have shown multiscale complexity analysis to be a good predictor of the variability of the signal over multiple time scales^22,23,26^. They have also shown that multiscale non-linear analysis may provide novel mechanistic insight into the extent to which the BF signal is modulated by the different frequency bands under differing (patho)physiological conditions^24,27^.

The primary objective of the current study was to investigate the complexity of the oscillatory rhythms in microvascular blood flow and tissue oxygenation signals obtained in the skin of individuals ascending to 5300 m as participants of the XE2 research expedition^28^, using non-linear uni- and multi-scale approaches. We hypothesised that non-linear approaches will provide novel information on the processes by which SH preserve superior peripheral microcirculatory perfusion at altitude^7–9^. We also sought to explore whether an enhanced complexity of the BF signal was associated with the more efficient oxygen delivery postulated to play a key role in the physiological adaptation of Sherpas to hypobaric hypoxia^5–8^.

Microvascular blood flow was measured non-invasively using laser Doppler fluximetry at two skin sites; that of the well characterised ventral forearm under both endothelium dependent and neurovascular control, and that of the finger pulp in which BF is largely determined by the abundantly present arteriovenous anastomoses under sympathetically mediated constrictor tone^29^. The analysis of signals from both beds allowed us to explore the differential impact of local flow-motion activity on the complexity of microvascular perfusion.

## Materials and Methods

### Study participants

The signals analysed are from measurements made in 32 lowlanders (LL) all of whom were born and lived below 1000 m, and were not from a native high altitude population, and 46 Sherpa highlanders (SH). These 78 individuals represent a subset of the 144 participants of the Xtreme Everest 2 research expedition (XE2)^28^ in whom we have previously reported the effects of hypobaric hypoxia on microvascular blood flow in the time and spectral domains^7^. Approval of the study design, risk management plan and protocol were obtained from both the University College London Research Ethics Committee (Ref: 3750/002) and the Nepal Health Research Council (NHRC) (1334). The study was performed to the standards set by the Declaration of Helsinki, except for registration in a database. All participants provided written informed consent for participation in the studies.

### Tissue blood flux and oxygenation signal capture

Skin microvascular blood flux (BF) and tissue oxygenation (OXY) signals, and skin temperature were recorded simultaneously at the forearm using a combined laser Doppler fluximetry (LDF) and white light reflectance spectroscopy probe (CP1T-1000 LDF™/OXY™/temperature probe, Moor Instruments Ltd, Axminster, UK) placed approximately 10 cm proximal to the wrist. A second combined LDF™/temperature probe (VP1T, Moor, Axminster, UK) was placed on the pulp of the index finger. Probe position was recorded with a photograph and permanent marker pen to ensure use of the same anatomical site in subsequent measurements as previously described^7^.

Signals were collected at baseline (BL) in London for LL (35 m), and in Kathmandu (1300 m) for SH, and at Everest Base Camp (EBC) (5300 m) as described previously^8^. Measurements were made on the morning of day 2 after arrival at EBC after an 11 day trek from Lukla (2800 m). Participants lay in a supine position during signal capture with their non-dominant arm resting at heart level. Full details of the environmental and physiological measurements made at each altitude are given elsewhere^7^.

### Signal analysis

All signals were captured at a 40 Hz sampling rate using the manufacturer’s software (moorVMS-PC software, Moor Instruments Ltd, Axminster, UK) and a continuous 10 min sample of each signal extracted for analysis in the time and spectral domain. The signals analysed were resting skin blood flux (BF, in arbitrary perfusion units, PU) and oxygenated Hb (oxyHb, in arbitrary units, AU). We elected to focus on the oxyHb output as the prime tissue oxygenation signal for the complexity analysis as suggested by our previous studies^22^.

The complexity of the BF and oxyHb signals arising from rhythmical flow-motion activity was explored using non-linear LZ complexity (LZC) analysis^21^ to determine how much the BF and oxyHb signals differed from a random sequence of finite symbolic sequences derived from the time series^20,30^. The signals were represented as a binary string using delta encoding whereby a zero is recorded if a value is less than the previous value in the time series and a one when it is greater than that previously^22,23^. The LZC is a measure of the information content, or effort to describe, a signal and is the length of the shortest instruction set required to reconstruct it without loss of information. A random signal would have high complexity as there are no rules that define it whereas a periodic signal would have low complexity as the same terms are repeated continually. LZC analysis does not require the signal to be strongly stationary, unlike chaos-based entropy analysis, and thus the signal can be normalised to the length of the sample window^31^. Exhaustive LZC, where the signal is decomposed into all the instructions required to reproduce it, was then calculated for each 40 second epoch to determine the complexity lower bound. A complexity index (LZC index) was calculated as the mean of the 15 × 40 second epochs for each sampled signal to provide an index of the dynamic activity modulating the BF and oxyHb signals.

The physiological processes that modulate flow-motion and determine the information content of the BF signals operate at frequencies ranging from 0.001 Hz to 2 Hz^14^. They also appear to vary with altitude^8,9^. To take account of these multiple, and potentially varying, process scales we measured LZC in multiple time-scales (MLZC) using a coarse-graining approach^24,32^. MLZC was explored between scales τ = 1–12, as the maximum frequency of interest is governed by an upper limit of heart rate of 1.6 Hz and Nyquist frequency twice this (3.2 Hz) which corresponds to scale τ = 12. To determine the association between the complexity of the BF signal and the mechanisms underlying flow-motion we calculated the Spearman correlations for relative power spectral density of the endothelial (0.0095–0.02 Hz), sympathetic (0.02–0.06 Hz), myogenic (0.06–0.15 Hz), respiratory (0.15–0.4 Hz), and cardiac (0.4–1.6 Hz)^14^ spectral bands and LZC measured at each time scale (MLZC).

### Statistical analysis

Statistical analyses were performed using SPSS for Windows version 25.0 (IBM, USA). Data were tested for Gaussian distribution using the Kolmogorov-Smirnov test and visual inspection of histograms. As the BF signal data did not show a normal distribution, data are presented as median (95%CI). SH and LL cohorts at BL and EBC were compared using two-way ANOVA. We used Mann Whitney U test and Wilcoxon Signed Rank test to compare single cohorts between BL and EBC. The relationships between BF, oxyHb and network perfusion complexity at each site were assessed individually using Spearman’s rank correlation coefficient. A p-value of < 0.05 was considered statistically significant for all analyses. We used multivariable linear regression models to explore factors that were independently associated, with microvascular network perfusion heterogeneity and oxygenation as the dependent (outcome) variables. Explanatory variables included in the models were skin temperature, age and gender, with site included as a binary indicator variable.

## Results

**Figure 1:**
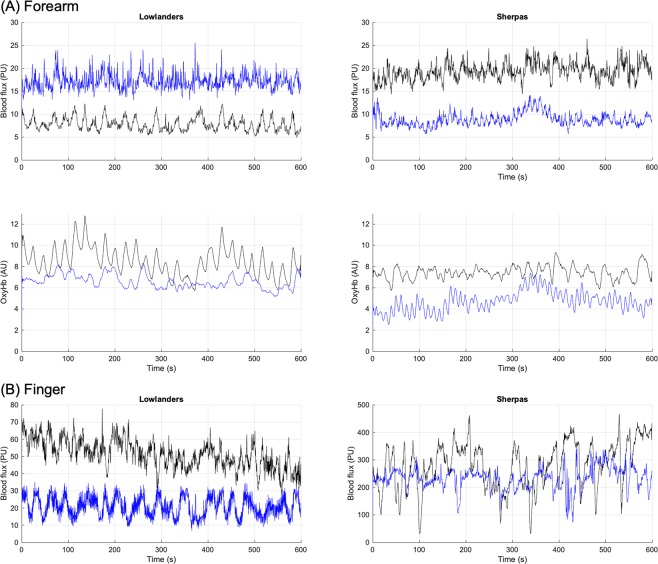
Examples of (**A**) skin BF and oxyHb signals from the forearm and (**B**) skin BF signals from finger measured in the time domain of an individual lowlander and Sherpa highlander at baseline (BL, black) and Everest base camp (EBC, 5300 m, blue).

Artefact-free skin BF and tissue oxygenation signals (Fig. 1) of sufficient length for LZC analysis at both BL and EBC, were obtained in 32 LL (16 M/16 F), age 46(14)y, BMI 24.3(3.3) kg/m^2^ (mean(SD)) and 46 SH (23 M/23 F), age 28(6)y (p = 0.0001, SH vs LL) and BMI 23.8(3.4) kg/m^2^ (p > 0.05, SH vs LL) who represented a subset of the 144 participants from the Xtreme Everest 2 research expedition (XE2)^28^.

The time domain characteristics of microcirculatory BF signals from the current subset of XE2 individuals used for complexity analysis are summarised in Table 1. Resting skin BF (PU) at the forearm of the 46 SH decreased with ascent to EBC, while that of the 32 LL remained unchanged, as reported previously in the full cohort^8^. In both sub-groups, resting BF at the finger decreased on ascent to EBC compared to BL (both p < 0.001). Skin temperature varied between groups and with ascent to EBC but was notably higher at the finger in SH at both BL and EBC (Table 1). In both groups resting BF were positively associated with skin temperature at BL at the forearm (LL, r = 0.370 p = 0.037; SH, r = 0.577 p < 0.0001) and at the finger (LL, r = 0.436 p = 0.013; SH, r = 0.764 p < 0.0001). This association was lost at EBC in LL (p > 0.05 for forearm and finger), but not in SH (forearm r = 0.261 p = 0.021; finger r = 0.643 p < 0.0001).

**Table 1:** Summary of peripheral oxygen saturation (SpO_2_) and skin blood flux and oxygenation recorded in the time domain at the forearm and finger in lowlanders (LL) and Sherpa highlanders (SH) at baseline (BL) and Everest base camp (EBC, 5300 m).

Median (95%CI)	Lowlanders N = 32		Sherpa Highlanders N = 46	
	BL	EBC	BL	EBC
SpO_2_ (%)	98.0 (98,99)	77.5*(74,81)	97.2 (97,98)	79.1* (78,80)
Skin BF forearm (PU)	11.5 (9.1,14.4)	14.1 (12.4,15.7)	15.2† (13.2,19.2)	11.2*† (9.2,13.1)
StO_2_ forearm (%)	40.9 (37.0,45.7)	27.1*(24.4,32.0)	36.3† (34.2,38.2)	28.4* (24.5,30.8)
oxyHb forearm (AU)	6.5 (5.6,8.9)	5.9 (4.6,8.3)	8.7† (7.3,10.6)	7.2* (5.8,8.7)
Skin BF finger (PU)	46 (26,84)	34 (21,52)	283† (247,327)	232† (178,278)
Skin Temp forearm (°C)	27.6 (26.8,28.2)	28.5 (27.6,29.5)	31.4† (30.6,32.7)	28.2* (27.2,30.7)
Skin Temp finger (°C)	23.9 (22.7,25.7)	23.7 (19.6,25.3)	32.0† (30.0,33.1)	28.2*† (23.6,30.5)

Data are median (95% confidence interval) from n = 32 LL and n = 46 SH. *BL vs EBC within group, ^†^LL vs SH within site. SpO_2_ (%) = peripheral oxygen saturation (%) (Nonin Onyx 9500, Nonin Medical Inc, Minnesota, USA); Skin BF (PU), = resting laser Doppler skin blood flux (perfusion units); StO_2_ (%) = tissue oxygen saturation (%); oxyHb (AU) = oxygenated haemoglobin (arbitrary units); Skin Temp (°C) = skin temperature (°C) (moorVMS-OXY, Moor Instruments Ltd, Axminster, UK).

Peripheral oxygen saturation (SpO_2_) declined with ascent to EBC in both LL and SH (p < 0.001), as did tissue oxygenation (StO_2_) measured at the forearm using VMSOXY (p < 0.001)^8^.

### LZ complexity of microcirculatory blood flux and oxygenation signals increases with hypobaric hypoxia

**Figure 2:**
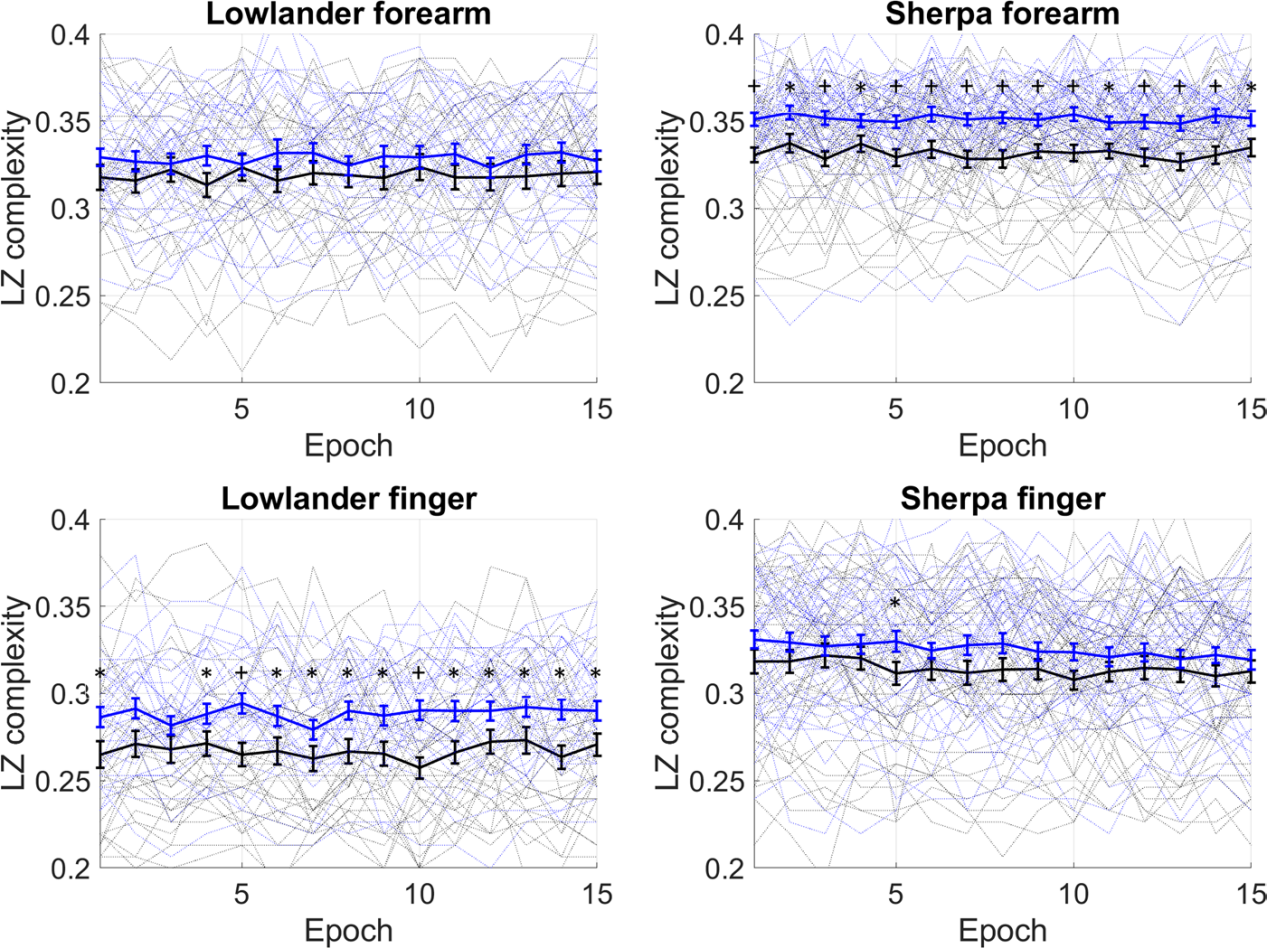
Lempel-Ziv complexity (LZC) of skin BF signal measured over 15 epochs at the finger and forearm at baseline (BL, black) and Everest base camp (EBC, 5300 m, blue). Mean data (±SEM) are shown as solid lines for n = 32 lowlanders and n = 46 Sherpa highlanders. Significant difference between groups, *p < 0.05, ^+^p < 0.001.

**Figure 3:**
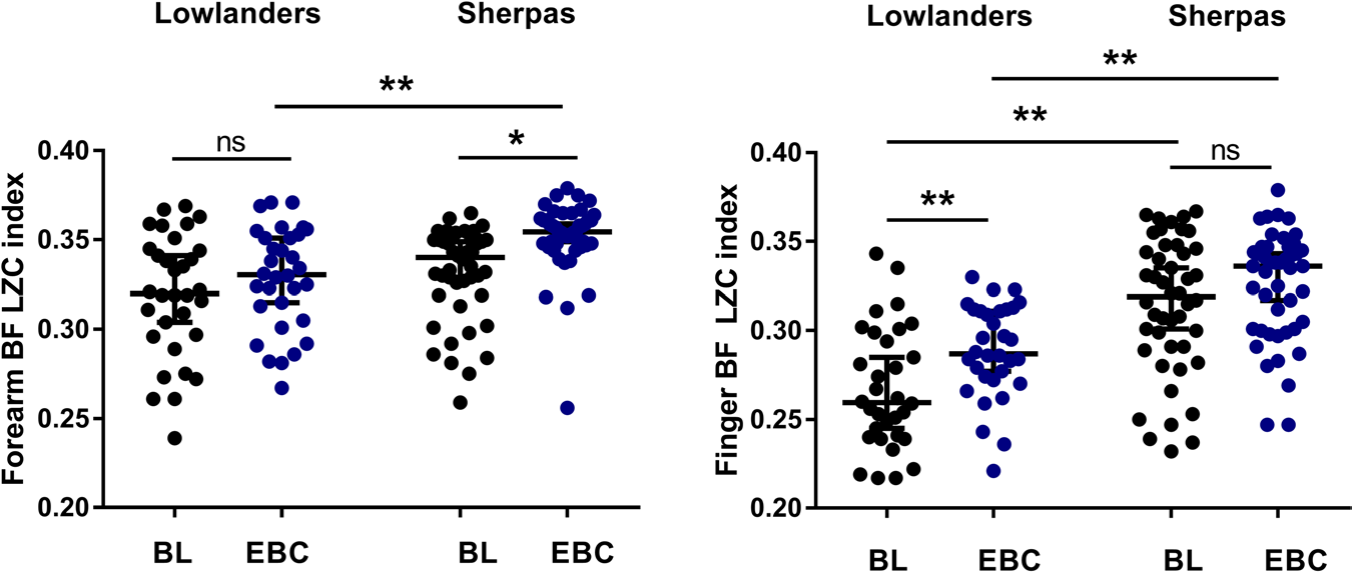
Lempel-Ziv complexity (LZC) index of skin BF signals measured at the forearm and finger in n = 32 lowlanders and n = 46 Sherpa highlanders at baseline (BL, black) and Everest base camp (EBC, 5300 m, blue). Data are median and 95%CI. LZC index (mean LZC of 15 epochs) of the BF signal in both the forearm and finger microvascular beds was significantly influenced by group (forearm: p = 0.0001, F = 15.6; finger: p < 0.0001, F = 59.9), and site (forearm: p = 0.0013, F = 10.7; finger p = 0.0025, F = 9.5). ns not significant, *p = 0.0003, **p < 0.0001.

The complexity of the BF and oxyHb signals arising from oscillatory flow-motion activity was determined using non-linear LZ complexity (LZC) analysis. Examination of the BF signals obtained from SH and LL at BL and on ascent to EBC, revealed that the information content of the BF signals remained relatively constant over the 15 epochs (600 s) analysed in both groups under normoxia and hypobaric hypoxia. As previously, it was thus deemed appropriate to additionally present the complexity of each signal as a mean LZC index^22,23^. Figure 2 shows that the complexity of resting BF signals measured at the forearm and finger in SH and LL remained relatively constant over the 15 epochs sampled, at both BL and EBC. LZC index (mean LZC of 15 epochs) of the BF signal in both the forearm and finger microvascular beds was significantly influenced by group (forearm: p = 0.0001, F = 15.6; finger: p < 0.0001, F = 59.9), and site (forearm: p = 0.0013, F = 10.7; finger p = 0.0025, F = 9.5). Forearm BF LZC index increased in SH with ascent to EBC (BL vs EBC p < 0.0031), and at EBC was significantly greater than that of LL (SH vs LL p < 0.0001) (Fig. 3**)**. There was no association between forearm BF LZC index and resting BF or skin temperature at BL or EBC in SH or LL.

LZC index of the finger BF signal was higher in SH than LL at both BL and EBC (both p < 0.0001), but only increased with ascent to EBC in LL (p = 0.0413) (Fig. 3). In SH, finger BF LZC index was positively associated with resting BF (p < 0.001 at both BL and EBC) and skin temperature at both altitudes (p = 0.001 at both BL and EBC). No such association was seen in LL.

Neither age nor sex were independently associated with BF LZC index in SH. However, in LL age was negatively associated with forearm BF LZC index (r = −0.491 p = 0.004).

**Figure 4:**
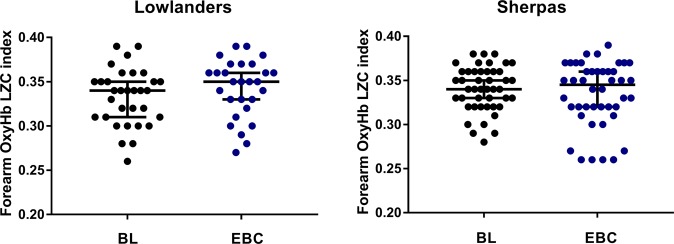
Lempel-Ziv complexity (LZC) index of skin oxyHb signals measured at the forearm in n = 32 lowlanders and n = 46 Sherpa highlanders at baseline (BL, black) and Everest base camp (EBC, 5300 m, blue). Data are median and 95%CI. There was no significant difference between groups.

We found no significant difference in LZC index of the oxyHb signals measured at the forearm between LL and SH, at either BL or EBC (p > 0.05) (Fig. 4). However, in SH forearm BF LZC index was positively associated with the LZC index of the oxyHb signal at both BL (r = 0.414 p = 0.005) and EBC (r = 0.307 p = 0.004). No such association was found in LL at BL (p = 0.672), and only approached significance at EBC (r = 0.353 p = 0.065).

### Changes in multiscale LZC (MLZC) at altitude are associated with flow-motion activity

**Figure 5:**
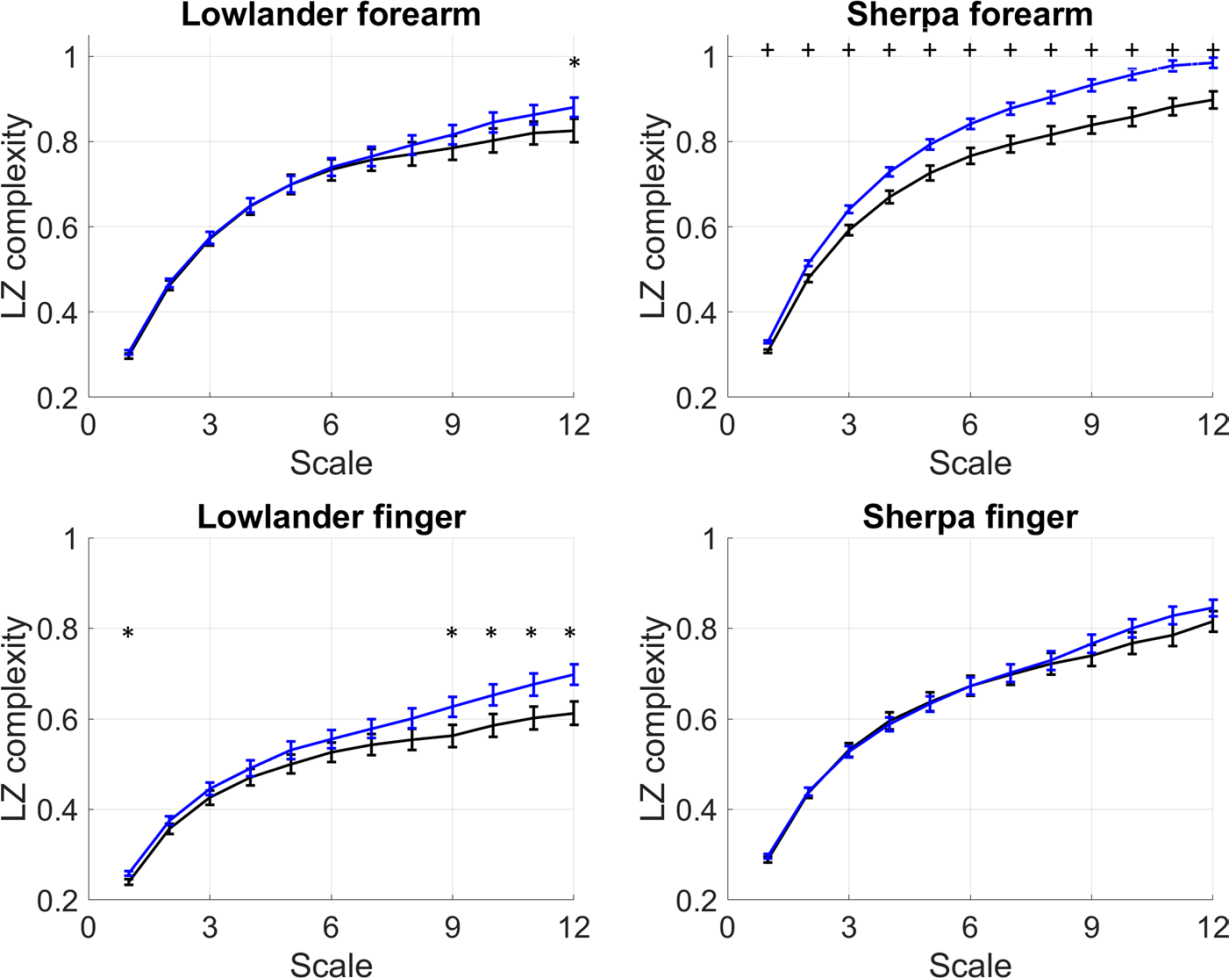
Multiscale Lempel-Ziv complexity (MLZC) measured between scales τ = 1–12 of skin BF signal at baseline (BL, black) and Everest base camp (EBC, 5300 m, blue). at the forearm and finger at baseline (BL, black) and Everest base camp (EBC, 5300 m, blue). Data are mean ± SEM of n = 32 lowlanders and n = 46 Sherpa highlanders. Significant difference between groups, *p < 0.05, ^+^p < 0.001.

The LZC of the BF signals at the forearm was computed over 12 scales for all LL and SH at BL and EBC (Fig. 5**)**. As the scale length increased, so too did LZC, with better separation seen at certain scales. In both SH and LL, the largest differences between the LZC values at BL and EBC were for time scales > 10 in both forearm and finger microvascular beds (p < 0.05).

**Figure 6:**
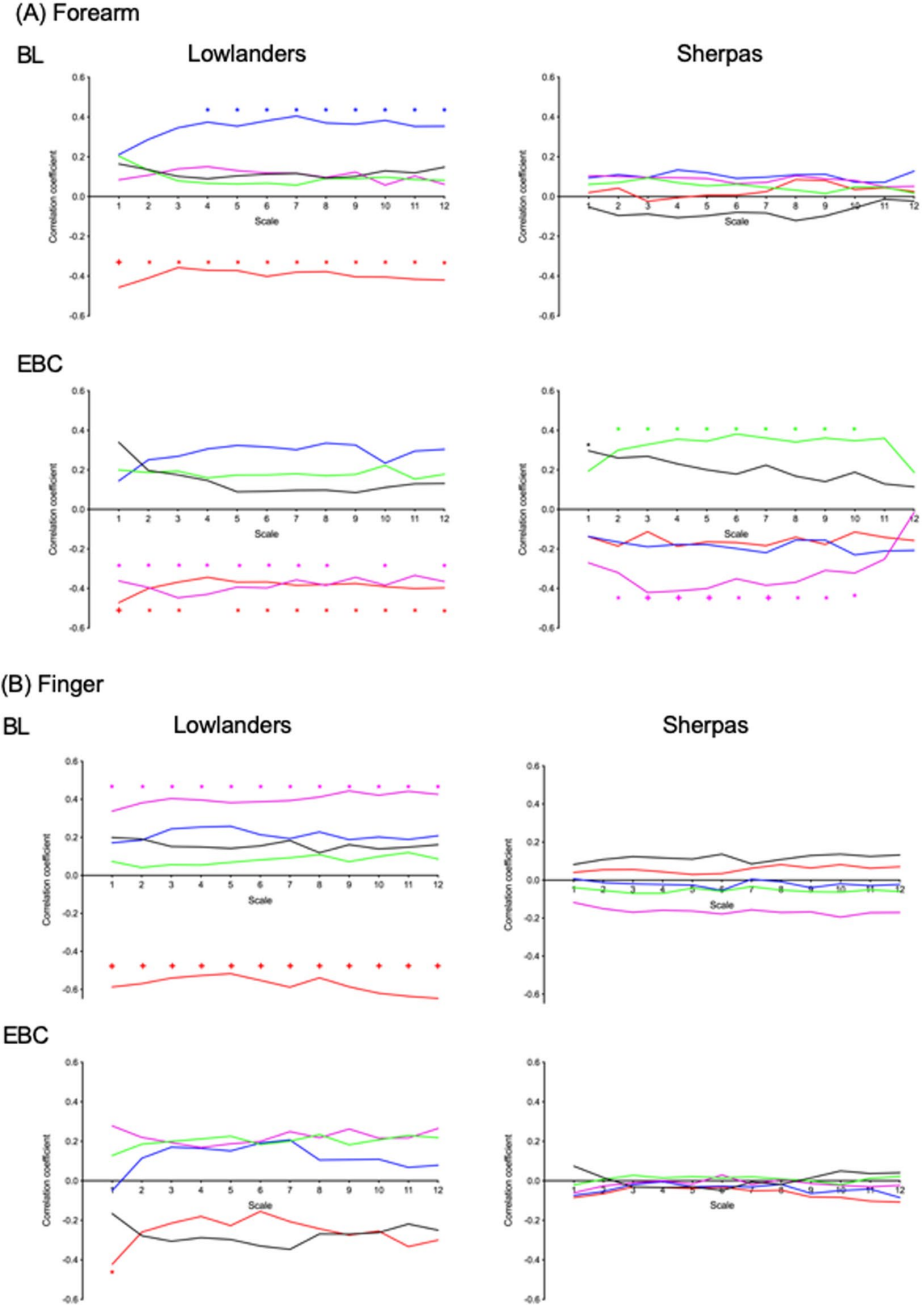
Spearman correlations between the flow-motion spectral power bands corresponding to endothelial (0.0095–0.02 Hz) (black), neurogenic (0.02–0.06 Hz) (green), myogenic (0.06–0.15 Hz) (purple), respiratory (0.15–0.4 Hz) (blue), and cardiac (0.4–1.6 Hz) (red) activity and multiscale Lempel-Ziv complexity (MLZC) of the (**A**) forearm and (**B**) finger skin blood flow signals in n = 32 lowlanders and n = 46 Sherpa highlanders at baseline (BL) and Everest base camp (EBC, 5300 m). Effective sampling rate = 40 Hz/scale. *p < 0.05, ^+^p < 0.01.

Figure 6 shows the association of the power in the five spectral bands to LZC forearm and finger BF complexity at each scale for the two groups at each altitude. There were clear differences in the associations between LZC and spectral power in the five bands both between SH and LL, and between BL and EBC in the forearm and finger BF signals. In LL, cardiac activity negatively associated with forearm BF LZC at all scales at both altitudes. Power in the respiratory band was positively associated with LZC over scales τ = 4–12 at BL, but did not reach significance at EBC. In the low frequency bands, the myogenic activity negatively associated with LZC at EBC over scales τ = 1–12 (40–3.34 Hz sample rate). No significant association was found between power in the neurogenic activity band  and LZC at any scale at either BL or EBC in LL. In SH, there was no association between LZC and spectral power in any band at BL. At EBC, there was a significant negative association between myogenic activity and forearm BF LZC, and a positive association between neurogenic activity and BF LZC across scales τ = 2–11. No association between forearm BF LZC and the high frequency activity bands was seen at either altitude in SH.

Similar trends were seen in the association between spectral power in the five bands and LZC in the finger BF signals. However, the only associations between BF LZC and spectral power that reached significance in the finger were in LL at BL, where cardiac activity negatively associated with BF LZC, and myogenic activity negatively associated with BF LZC at all scales (Fig. 6).

## Discussion

We have shown that the LZ-complexity of the skin microvascular blood flux signals, measured in participants of the XE2 research expedition^28^, increased with ascent to EBC (5300 m). We observed that SH exhibited a greater level of network complexity and hence capacity for heterogeneous flow distribution than LL, at both BL and EBC. We also found clear differences in the influence of local flow-motion activity on the information content, and hence complexity of the BF signal, between SH and LL on ascent to altitude. Finally, we have shown that in SH, the increased complexity of microvascular network perfusion is associated with an enhanced complexity of the simultaneously sampled oxyHb signals, suggestive of an improved ability in SH to match O_2_ demand to local O_2_ delivery under conditions of hypobaric hypoxia.

We have previously shown using time and spectral domain analysis of BF signals that SH, when exposed to hypobaric hypoxia, demonstrated superior preservation of peripheral microcirculatory perfusion compared to LL and that in SH differences in local myogenic (vasomotor) and neurogenic control may play a key role in their adaptation to high altitude by sustaining local perfusion and tissue oxygenation. However, a robust and consistent mechanistic description of flow dynamics within the microcirculation cannot be achieved using time domain analysis (in either the resting state or during haemodynamic perturbation) or frequency domain analysis methods alone^33^.

Non-linear complexity-based analysis applied to BF signals derived from the peripheral vasculature has been shown to yield additional, and much deeper, understanding of the loss of system flexibility, and to enhance risk assessment^24,26^. In the current study, LZC index was higher in SH than LL in both forearm and finger BF signals (Figs 2 and 3). It also increased to a greater extent in SH than LL on ascent to altitude. LZC of the BF signal has previously been shown to decline in individuals with or at risk of CVD^24^ and in primates with the onset of diabetes^27^. Similarly, Frisbee *et al*.^32^, using chaotic network attractor analysis to explore the spatial and temporal shift in perfusion distribution at successive arteriolar bifurcations within skeletal muscle, have shown an imbalanced and temporally stable distribution of flow through the microvascular network in rodent models of increasing metabolic and CV disease risk. Our current findings suggest that the increase in variability in the BF signal seen in SH is indicative of a beneficially enhanced microcirculatory adaptive capacity through more effective autoregulation within the microvascular network^22,34,35^.

Distributive alterations and heterogeneity of flow within microvascular networks are critical to an adequate tissue oxygenation^10,11^ and oscillatory fluctuations in microvascular network flow have been shown to ensure a more effective tissue oxygenation than would be obtained with a steady blood flow^16^. While we report no change in LZC of the oxyHb signal with ascent to EBC nor differences between SH and LL, we did observe a positive association between the LZC index of forearm BF and LZC index of oxyHb signals in SH, measured simultaneously at the forearm, at both BL and EBC. We additionally found that in SH the enhanced complexity of network perfusion measured at both altitudes was positively associated (r = 0.252 p = 0.019) with the previously reported microvascular oxygen unloading rate, measured in the same individuals^8^. Such a relationship was not seen in LL (p = 0.761). The distribution of O_2_ in tissue depends on microvascular network structure and flow and haematocrit distributions, which are all markedly heterogeneous^10^. Disturbed capillary flow patterns have been shown to limit the efficacy of oxygen extraction even in the absence of changes in mean flow^36^. While we are unable to demonstrate a causal relationship between microvascular blood flow, complexity of the perfusion signals and tissue oxygenation, such a relationship would appear consistent with a beneficial adaptation in SH whereby enhanced variability in flow-motion activity gives rise to more effective O_2_ unloading.

The increase in LZC index derived from laser Doppler BF signals from the skin with ascent to EBC, and the differences in variability of the signals between SH and LL, appear at first to be contrary to the decrease in flow heterogeneity index (HI) reported by Gilbert-Kawai *et al*.^7^ derived from direct observation of the movement of red blood cells in the buccal mucosal microcirculatory bed using incident dark-field imaging. In this study of 64 SH and 69 LL from XE2, Gilbert-Kawai *et al*. report that in SH sublingual blood flow increases on ascent to high altitude in a ‘’uniform and homogenous manner” in vessels < 25 µm diameter. By contrast they report that in LL microvascular flow decreased, but observed that this decrease was “not in a uniform manner, such that it became heterogeneous in nature”. HI is calculated using a semi-quantitative technique, as the highest site flow velocity minus the lowest site flow velocity divided by the mean of the flow velocities across all sublingual vessels imaged^37^. HI is thus indicative of variations in red blood cell flow velocities across a series of individual capillaries. By contrast, LZC index is a measure of the algorithmic complexity of the time series data of the laser Doppler blood flux signal and yields a measure of the variability or predictability of the oscillatory signal. As such, these two indices cannot be compared. It should not be neglected that capillary density has been reported to increase under conditions of hypobaric hypoxia^1^, and that while Gilbert-Kawai *et al*.^7^ report that sublingual small vessel density was not different between the SH and LL at BL testing, capillary density was up to 30% greater in SH at EBC. Taken together, these data from the buccal mucosa suggest that SH maintain a significantly greater microcirculatory flow per unit time and flow per unit volume of tissue than LL at high altitude; an anticipated consequence of which might be greater information content of the BF signals and consequently a higher LZC.

From a signal perspective, variability in BF arises from the cumulative activity of all the processes modulating BF and their temporal variation. Fluctuations in microcirculatory flow occur at different frequencies related to local endothelial (0.0095–0.02 Hz), sympathetic (0.02–0.06 Hz) activity, myogenic activity in the vessel wall (0.060.15 Hz), and modulation by respiratory (0.15–0.4 Hz), and heart (0.4–1.6 Hz) rates^14^. Examination of the information content of the BF signals from SH and LL revealed a clear and significant different in LZC between the two groups on ascent to altitude that becomes more pronounced at certain timescales (or sampling rates). Furthermore, by examining the association of the individual spectral bands associated with flow-motion with the different time-scales in MLZC, our data provide strong evidence that the influence of the modulators of flow-motion activity differs between SH and LL and with ascent to altitude. The Spearman correlations between the power bands of the forearm BF signals and LZC across multiple time scales shown in Fig. 6 showed that in LL there was a significant contribution of cardiac power compared with SH, at both BL and EBC. Consequently, the BF signal had proportionally more periodic content in LL and the complexity of the signal was reduced as the information content falls. Previous studies in altitude-naive LL and high altitude residents have shown that autonomic function, heart rate variability and respiration rate are differentially affected at high altitude, with LL showing sympathetic activation to modulate the direct vasodilatory effects of hypobaric hypoxia^38,39^. Heart rate variability is also known to contribute to complexity of the BF signal^40,41^ and cardiac rhythm to be modulated by respiratory oscillation^42^. This coupling of the two HF components offers a possible partial explanation as to why the MLZC increases with scale in both SH and LL (Fig. 5).

The lower frequencies associated with flow-motion (that generally contain most of the power in the signal) also contributed to signal variability. We report a marked uprating of the association between neurogenic power with LZC of BF measured at the forearm at high altitude, which is positively associated in SH. This appears consistent with the preservation of vasoconstrictor response and enhanced neurogenic activity reported in SH at altitude reported previously^8^. Such changes are indicative of an adaptive modulation of sympatho-vagal activity through which SH can better regulate flow, allowing them to stay in a hypobaric atmosphere at lower temperatures without excessive autonomic stress^43^. These adaptations may also contribute to the higher skin temperatures measured in SH. We saw no significant uprating of the association between neurogenic power and LZC of BF at any scale measured at the forearm of LL at high altitude.

Myogenic activity (vasomotion) is closely associated with effective oxygen delivery^17,44,45^ and has been shown to increase in hypobaric hypoxia^8,9^. Consistent with this we observed an increased association of the myogenic power band with LZC of the blood flux signal over multiple time scales in both SH and LL, under conditions of hypobaric hypoxia at EBC. The association of myogenic power with BF LZC was negative, consistent with its periodic nature. While the effect of flow-motion on the transport of oxygen to tissue ‘is highly complicated’^46^, an increase in the lower frequencies of flow-motion and particularly in vasomotion has been shown to give rise to transients in the partial pressure of O_2_ (PO_2_) to substantially increase the volume of oxygenated tissue and to oxygenate tissue domains which under steady-state conditions would remain anoxic^11^. Indeed, mathematical modelling suggests that vasomotion activity can change oxygen delivery to tissues by up to eightfold under certain conditions^47^. Clinically, it has been shown that vasomotion is increased in patients with mild peripheral arterial occlusive disease (PAOD) and that those patients with enhanced vasomotion had significantly higher tissue oxygen levels than those without, despite similar blood flow^48^. Our data are also consistent with the increase in vasomotion seen in reduced perfusion states such as sepsis and cardiopulmonary bypass and in multiple organ dysfunction and mortality^49–51^. Thus, the increased strength of the association of the myogenic power band activity with LZC, is consistent with vasomotion being an important modulator of gaseous exchange under conditions of hypobaric hypoxia, when the microcirculation may tend towards a critical state.

We found little association between forearm BF LZC and the power of the endothelial frequency band in either LL or SH. While the strength of the association in SH was greater at EBC than at BL, it only reached significance at the highest sampling frequency of 40 Hz (τ = 1). Thus, while endothelium-mediated flow-motion might be expected to enhance network perfusion and tissue oxygenation, we can as yet draw few conclusions on the role of the endothelium-attributed flow-motion activity within the skin microvasculature at altitude.

This study has several strengths, not least the large and sex balanced group size. While we saw no independent effect of sex or BMI on our outcome measures of complexity in either SH or LL, we did observe a negative effect of age on BF LZC index in LL but not in SH. LL (mean age 46(14)y) were significantly older than the SH (28(6)y) (p = 0.0001, SH vs LL) in this subset of participants from XE2, and it is probable that increasing age constitutes a risk factor for declining heterogeneity of microcirculatory network perfusion^52^. A similar age-related reduction in signal complexity has been reported using nonlinear measures applied to cardiac signals^53^ and signals derived from large blood vessel (pulse wave velocity)^15^.

To the best of our knowledge, this study is the first to analyse the effect of hypobaric hypoxia at high altitude on the microcirculation using complexity-based measures applied to skin microvascular LD blood flux signals. We have previously demonstrated that complexity-based measures can differentiate both between haemodynamic states^22,23^ and between groups of individuals at increasing risk of developing microvascular dysfunction^24^. The current study extends and strengthens the utility of these approaches to widely varying cohorts.

We analysed signals from two skin sites that allowed us to explore the differential impact of local flow-motion activity on network flow heterogeneity. Skin is a major thermoregulatory organ sensitive to environmental temperature. The temperature of the London laboratory where baseline measurements were performed in lowlanders was lower than that in Kathmandu where Sherpas were studied. It is therefore probable that this contributed to the lower forearm and finger fluxes seen in lowlanders at baseline. While both cohorts were exposed to the same laboratory conditions at EBC, finger BF and skin temperature remained significantly higher in SH than LL. This suggests that the physiological differences in SH and LL seen at EBC are independent of external temperature. However, the variations in skin temperature across the cohorts and altitudes may be expected to differentially influence relative flow-motion activity and signal complexity^23^ as will differences in haematocrit that have been reported previously^5–7^ and which may influence the laser Doppler signal^8^.

The paucity of significant associations between LZC and spectral power across the five frequency bands measured at the finger was unexpected, as LZC of the BF signals measured at both the forearm and the finger were higher in SH than LL and both influenced by ascent to altitude. It was also unexpected as skin BF at the finger is largely determined by arteriovenous anastomoses under vasoconstrictor sympathetic control^29^ and we have previously shown that ascent to EBC results in differential changes in vasoconstrictor responses and in local flow-motion activity in the low frequency bands in the resting blood flux signals in SH and LL^8^.

In exploring the associations between LZC and spectral power of the BF signals, we used fixed non-overlapping intervals to define the spectral bands as described previously by Stefanovska and colleagues^14^. It is unlikely that the boundaries of these frequency intervals remain constant across a cohort of individuals, or for a given individual, under changing conditions of physiological stress. State-dependent fluctuations in frequency intervals may thus give rise to different patterns in complexity within, and across, the cohorts studied. It would be interesting to explore further the impact of time-varying spectral band boundaries on signal complexity, and how this may inform a mechanistic interpretation of the heterogeneity of microvascular network perfusion.

## Conclusions

These data confirm that synchronicity of rhythms in the modulators of microcirculatory blood flux signals, assessed using non-linear complexity analysis, contributes to the heterogeneity of microvascular perfusion. They also go some way to describe the changes in the activity of local and systemic physiological mechanisms that modulate the potentially beneficial adaptation response seen in SH under conditions of hypobaric hypoxia. Together these data suggest that peripheral tissues play an important physiological role in the cardiovascular adaptation to hypoxia, and that this role is better developed in native altitude dwellers than in lowlanders.

In future, it is possible that a combination of time, frequency and complexity analysis will yield a deeper understanding of the loss of system flexibility that may prevent the microvascular networks from adapting to an imposed stressor and provide further insight into the parameters that influence this.

## Data Availability

Data supporting this study are openly available from the University of Southampton repository at 10.5258/SOTON/D1076.
